# The effect of practical cooling strategies on physiological response and cognitive function during simulated firefighting tasks

**DOI:** 10.15171/hpp.2017.13

**Published:** 2017-03-05

**Authors:** Rasoul Hemmatjo, Majid Motamedzade, Mohsen Aliabadi, Omid Kalatpour, Maryam Farhadian

**Affiliations:** ^1^Department of Occupational Health, School of Public Health, Hamadan University of Medical Sciences, Hamadan, Iran; ^2^Department of Ergonomics, Hamadan University of Medical Sciences, Hamadan, Iran; ^3^Department of Occupational Health, Hamadan University of Medical Sciences, Hamadan, Iran; ^4^Department of Biostatistics, Hamadan University of Medical Sciences, Hamadan, Iran

**Keywords:** cognitive function, cooling strategies, firefighting, physiological responses, smoke-diving

## Abstract

**Background:** Firefighters often perform multiple tasks during firefighting operations under unknown and unpredictable conditions in hot and hostile environments.

**Methods:** In this interventional study each firefighters engaged in 4 conditions: namely (1) no cooling device; control (NC), (2) cooling gel (CG), (3) cool vest (CV), and (4) CG+CV. Cooling effects of the employed interventions were evaluated based on heart rate (HR), temporal temperature (TT), reaction time (RT), and the correct response (CR).

**Results:** HR and TT values for use of CG+CV (147.47 bpm [SD 4.8]; 37.88°C [SD 0.20]) and CV bpm (147.53 [SD 4.67]; 37.90°C [SD 0.22]) were significantly lower than the CG (153.67 bpm [SD 4.82]; 38.10°C [SD 0.22]) and NC (154.4 bpm [SD 4.91]; 38.11°C [SD 0.23]) at the end of the activity. RT and CR for use of CG + CV (389.87 ms [SD 6.12]; 143.53 [SD 1.24]) and CV (389.53 ms [SD 6.24]; 143.47 [SD 1.18]) were significantly higher than the CG (385.73 [SD 7.25] ms; 143.07 [SD 0.88]) and NC (385.67 ms [SD 7.19]; 143.00 [SD 0.84]) at the end of the activity.

**Conclusion:** It is concluded that CV was more effective than the CG in attenuating physiological responses and cognitive functions during firefighting operations. Furthermore, combining CV with CG provides no additional benefit.

## Introduction

Firefighters are employed in an occupation in which they are exposed to various stressors (e.g. night shift schedules, sudden alarm calls, strenuous physical work, exposure to smoke and rescue operations) for an unpredictable amount of time.^1-5^ The various stressors that firefighters deal with cause stress, which entails the body’s reaction to a particular event.^6,7^ The effect of stress on cognitive function is assessed through investigating the body’s reaction to stress. When the stress level rises during firefighting operations, the body deals with stress, an issue that may reduce the capacity of information processing and decision-making.^8^This is due to the fact that the stress hormones secreted as a result of various stressors are steroids and they can easily overcome the blood–brain barrier and reach the brain. They can then impact learning and memory by binding to receptors located in various brain regions which are involved in learning and memory.^6^ Some researches have noted that impairment of cognitive function can increase the risk of injury and death.^9^

Fire service workers usually perform their duties in hot and hostile environments while wearing thick and heavy clothing.^8,10,11^It is recognized that working in a strenuous environment creates greater physiological strain and cognitive function impairment than working under thermoneutralcondition.^12-15^ In addition, studies have reported greater cardiovascular and thermoregulatory strains when working in protective clothing rather than in normal clothing.^16,17^According to previous studies, the specific characteristics of protective clothing of firefighting (i.e. being heavy, thick, massive, and laminated) limit the degree of water evaporation and therefore, increase metabolism.^8,18^Other studies suggest that these factors cause disturbances in the physical performance and can also cause changes in the firefighters’ cognitive function.^19^

Considering the relation among firefighters’ protective clothing, strenuous environments, and the well-documented cognitive function impairment and physiological strain with firefighting activities, there is a requirement to develop methods for keeping firefighters’ cognitive function and physiological strain below critical levels during work in protective clothing. Work and rest schedules can be performed to prolong operations beyond those carried out in a continuous way. However, it has been reported that, with hot or humid conditions, body core temperature will not decline during passive rest, and, in fact, will maintain in excess during the schedule’s rest periods due to the environmental conditions.^11,17^

The effects of cooling strategies on the physiological responses in firefighters during moderate-intensity activities are well documented.^11,20,21^ There are several methods for cooling, including liquid and air cooling systems, water immersion of hands and feet, neck cooling, ice vest, and application of menthol for cooling.^20,22-25^ Studies have demonstrated that hand and forearm immersions and cool vests (CVs) are effective methods in reducing physiological strain following practice in firefighting protective clothing.^11,20,26^ Previous studies have shown that forearm immersion during the recovery period is more effective than ice vests in reducing physiological strain.^21^ However, during firefighting operations, it is not practical to cool firefighters’ bodies through strategies like hand and forearm immersion and head washing since it is not feasible for firefighters to take off their clothing and personal protective equipment. In addition, previous study disclosed that ice vests are more effective at lowering heart rate (HR) and skin temperature during exercise in the heat.^27^ Therefore, CVs and CG are more practical during firefighting activity.

Previous researches have disclosed that simulated firefighting has an effect on cognitive function.^9,12,28^ It has also revealed that neck cooling enhances performance and reduces the number of search errors made during the search and memory test.^24^ Hence, in order to perform their tasks perfectly and prevent damages caused by fire suppression, firefighters must have certain physiological, physical and cognitive ability.

As one of the first attempts, this experimental study investigated the effect of practical cooling strategies on both physiological response and cognitive function during simulated firefighting tasks in the advanced smoke-diving room. The present study was designed to:

1. Assess the effect of typical firefighting tasks and cooling devices on physiological response and cognitive function capacity during simulated firefighting activity‏.
2. Introduce an effective and practical cooling strategy for reducing the physiological and cognitive function impairment associated with firefighting activity in the hostile environment.

## Materials and Methods

### Participants

Fifteen healthy male firefighters, working at Iran’s National Petrochemical Company (NPC), were recruited for this interventional study, which was approved by the Ethics Committee of Hamedan University of Medical Sciences. Each firefighter was informed about the experimental procedure and purpose of the study. Before conducting the study, subjects’ health status in terms of the thermoregulatory and cardiovascular conditions as well as mental conditions were monitored by reviewing their medical records. Based on the results of this checkup, healthy firefighters were recruited. They undertook simulated firefighting activities in four experimental conditions (each experimental condition lasted for at least 45 minutes). All participants signed a consent form. The mean physical characteristics of the firefighters were as follows: age 32.47 (SD 5.96) years, height 1.79 m (SD 0.06), weight 82.8 kg (SD 15.08), body mass index 25.43 kg m^-2^ (SD 3.42), and body surface area 2.03 m^2^ (SD 0.20).

### Smoke-diving room

**Figure 1:**
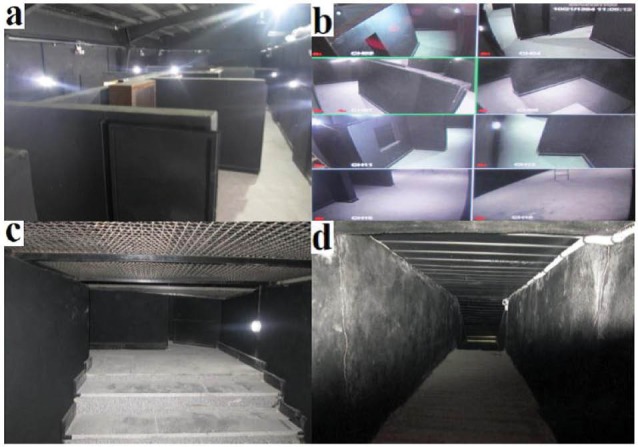
An overview of the smoke-diving room (a), control room (b), very narrow routes for crossing (c), escape tunnel (d).

In the current study, the firefighters conducted simulated firefighting activities in a smoke-diving room, which is an indoor environment with dark and nested rooms. It is used for exercising firefighting activities. Smoke-diving rooms are different in terms of their facilities (e.g. nesting rooms, heating and cooling systems, and specialized UV cameras), design and physical space. The smoke-diving room that was used in the current study was a 3000 m^2^ indoor space with black walls. Inside of the designed room, some of the walls, windows, very narrow roots for firefighters’ crossing and escape tunnels had been installed (Figure 1). In this room, a separate partition, named the control room (12 m^2^ indoor space), was constructed and control devices (e.g. monitors) were placed inside it. The temperature of control room was adjusted using a heating and cooling system (wet-bulb globe temperature [WBGT] 22°C, 50% relative humidity [RH]). Furthermore, advanced UV cameras were installed in various parts of the smoke-diving room to keep firefighters’ activities under surveillance.

Since the smoke-diving room was very large, it was very difficult to control its temperature and humidity‏. However, using several adjustable heating and cooling devices in different parts of the smoke-diving room, we were rather successful in controlling temperature and humidity (WBGT 28-30°C, 55%-60% RH)‏. It should be noted that a calibrated WBGT meter (CASELLA Instruments) was used to measure WBGT as an acceptable index of heat stress. Based on ISO7243, the WBGT index involves weighting of the dry-bulb temperature (Tdb), the natural wet-bulb temperature (Tnw) and the black-globe temperature (Tg).^29^ Artificial smoke and steam (deionized water) generators were used in this study to create fog in some parts of the smoke-diving room, hence simulating a real environment. Compared to other studies, examining the effect of simulated firefighting activities on firefighters’ physiological and cognitive functions, in the present research, we had significantly better facilities in terms of the physical space and available resources (e.g. advanced, specialized UV cameras).

### Continuous performance test 

The aim of continuous performance test (CPT) is measuring the capacity of cognitive function.^30,31^CPT, a principal test made by Rosvold et al,^32^ to survey vigilance, was used to take quantitative information regarding an individual’s capability to maintain attention. This task assesses both the speed of responding (i.e. reaction time, RT) and also the accuracy of responding (i.e. correct response, CR). In line with previous studies, in order to conduct the CPT, the firefighters were requested to sit in a 15–24-inch distance from the computer monitor, with the center of the monitor being 1–2 inches below eye level. An ergonomic mouse, which placed in front of the computer screen, was used to record participants’ responses; that is, they should press the left button of the mouse. Firefighters were instructed to respond to certain target stimuli continuously for a sustained period of time. Stimuli consisted of ten pictures which were presented successively for 200 ms on a computer monitor. The target stimulus (i.e. a star shape) was yellow in color, which appeared with only 20% frequency. The firefighters’ task was to decide, as quickly as possible, whether the picture was a star shape. The subjects were instructed to respond by immediately pressing a button when star shape was presented. The test lasted about four minutes and consisted of 150 stimuli.

### Experimental design

**Figure 2:**
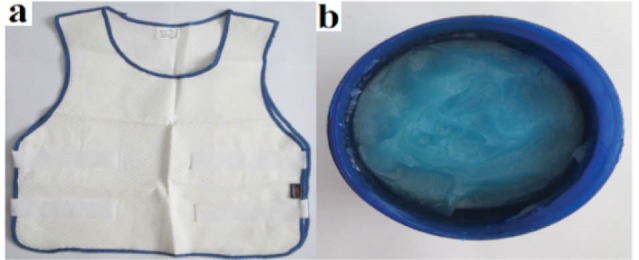
Outlast cool vest (Microgard, UK) (a), cooling gel containing menthol (b).

To assess the effects of cooling strategies and strenuous firefighting activity on physiological and cognitive function, all firefighters visited the smoke-diving room on four separate occasions, namely (1) no cooling device; control (NC), (2) cooling gel containing menthol (CG), (3) CV, and (4) CG and CV (CG+CV), in a random order. The CG (Golafshan, Iran) was used in the neck and forehead and, based on Lee et al,^33^ reports the amount of CG coated over neck and forehead was on average 0.77 ± 0.20 g/6.25 cm^2^ in body surface area. The CV (Microgard, UK), which was very light, was worn over a cotton T-shirt and was 100% polyester (PES) fabric coated with phase change material (PCM capsules) (Figure 2). Throughout the 4 experiments, subjects wore firefighting protective clothing (FPC) and basic undergarments: A T-shirt, underwear, socks, boots, protective gloves, protective helmet and self-contained breathing apparatus (SCBA) were donned. The total weight of the equipment was 22 to 26 kg. In this study, the practical cooling strategies were applied during firefighting activities while firefighters were wearing protective clothing.

Because the smoke-diving room should be built and equipped with the required equipment (e.g. specialized UV cameras as well as heating and cooling systems), it took a long time to execute the project. Nevertheless, firefighters were prepared for the implementation of the simulated activities in about one month and a few days. During this time, the firefighters were trained how to perform simulated firefighting operations correctly and run a CPT test. Therefore, before conducting the experiments, the participants attended practice meetings in which they got familiar with the procedure for taking the simulated firefighting activities in the smoke-diving room (WBGT 28-30°C, 55%-60% RH). As well as, cognitive test practice meetings were administered to familiarize participants with the CPT. It should be noted that training firefighters for the CPT test continued until firefighters were able to take part in the CPT test using the provided equipment, including computer and the installed software program, without the researchers’ assistance. In general, the implementation of the CPT for each firefighter was a very simple task and just included pressing a button upon seeing the target stimulus on the computer screen.

**Figure 3:**
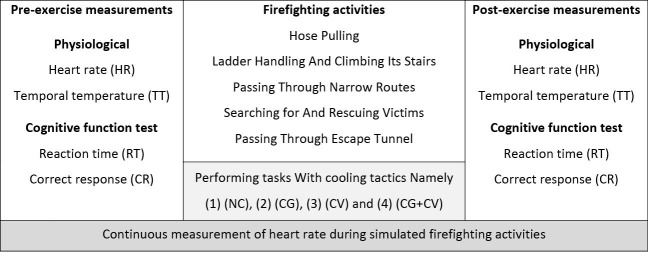
Protocol timeline of simulated activities with cooling strategies and measures during the period of the examination.

According to the protocol, the firefighters should accomplish the determined firefighting tasks in the simulated smoke-diving room. All firefighters performed four separate conditions in the smoke-diving room as quickly as possible. After entering the control room, each firefighter wore a HR sensor, and their basic physiological parameters were measured after 15 minutes of rest in the control room. Then, they performed CPT in the control room before entering the smoke-diving room. Subsequently, using different body cooling strategies, each firefighter performed defined simulated firefighting activities (e.g. hose pulling, ladder handling and climbing its stairs, passing through narrow routes, search and rescue operation, and passing through escape tunnel) (Figure 3). After that, they again performed CPT in the control room. They were asked to perform the drill intently and without competing. In The current study, firefighting activities were selected in the light of the previous studies.^2,12,34^ It took about 45-50 minutes for each firefighter to finish all simulated firefighting activities and the CPT.

It should be noted that HR was measured prior to the experiment in the smoke-diving control room and every minute throughout the trial using an HR monitor (Polar V800, Finland). In order to monitor the heart beat every minute, subjects wore a polar HR sensor. Before the experiment, firefighters needed to pair the HR sensor with HR monitor. Temporal artery temperature was measured prior to testing and at the end of each experiment using an infrared thermometer (ROSSMAX HC700, Switzerland).

### Statistical analyses 

The collected data were analyzed by SPSS 21 (SPSS Inc., Chicago, IL, USA). The Kolmogorov-Smirnov test was used to assess the normality of the data. Firefighters’ physiological and cognitive function response before and after exercise in the four experiments were compared using a series of paired samples *t* tests. The effect of cooling strategies on all measurements was tested by a repeated measures analysis of variance (ANOVA) for each of the dependent variables to determine if there were any significant differences during firefighting activities. The statistical significance was set at 0.05.

## Results

### Physiological responses

**Figure 4:**
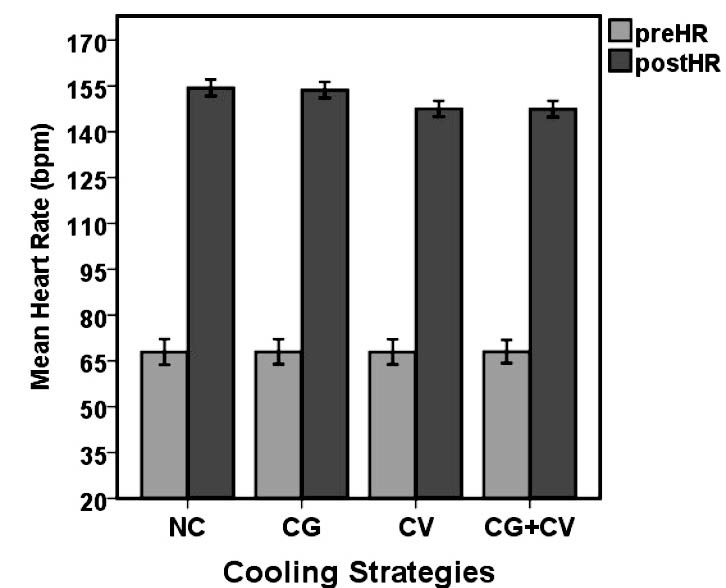
Comparison of the mean HR between the before (preHR) and after (postHR) activity in the 4 experimental conditions (*P*<0.05).
Abbreviations: NC, no cooling device; CG, cooling gel contain menthol; CV, cool vest; CG+CV, cooling gel and cool vest.

Figure 4 presents the HR responses during firefighting tasks. As expected, post HR (HRpost) was remarkably higher than the baseline (HRpre) during simulated firefighting activities throughout the exercise periods. Before and after the four experimental conditions, HRs were (67.93 bpm [SD 7.70] vs. 154.4 bpm [SD 4.91]), (68.00 bpm [SD 7.44] vs. 153.67 bpm [SD 4.82]), (67.93 bpm [SD 7.47] vs. 147.53 bpm [SD 4.67] ), (68.07 bpm [SD 6.84] vs. 147.47 bpm [SD 4.80] ) for NC, CG, CV and CG+CV, respectively. The results of paired samples *t* tests showed a significant difference in HRs between the beginning and the end of the simulated firefighting tasks in the four experimental conditions (*P*<0.05).

**Figure 5:**
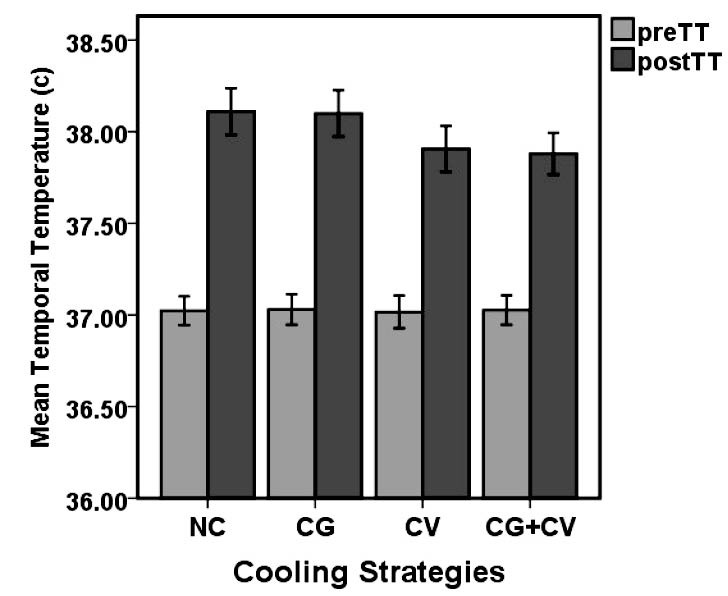
Comparison of the mean TT between the before (preTT) and after (postTT) activity in the 4 experimental conditions (*P*<0.05).
Abbreviations: NC, no cooling device; CG, cooling gel contain menthol; CV, cool vest; CG+CV, cooling gel and cool vest.

The mean temporal temperature (TT) scores for firefighters before and after exercise in the 4 experiments is presented in Figure 5. As shown in this figure, mean TT scores were remarkably different between pre- and post-experiment throughout the exercise periods. Before and after the firefighting activities, mean TT values were (37.02°C [SD 0.14] vs. 38.11°C [SD 0.23]), (37.03°C [SD 0.15] vs. 38.1°C [SD 0.22]), (37.01°C [SD 0.16] vs. 37.9°C [SD 0.22]), (37.02°C [SD 0.14] vs. 37.88°C [SD 0.20]), for NC, CG, CV and CG+CV, respectively. The paired samples *t* test revealed a significant difference in TTs between the beginning and the end of the simulated firefighting tasks in the smoke-diving room (*P*<0.05).

Pairwise comparisons of cooling impacts on physiological responses in the 4 experiments are displayed in Table 1. A repeated measures analyses of variance was used for pairwise comparisons to examine the effect of cooling strategies on the physiological responses. As Table 1 indicates, there is no significant difference in HR and TT between NC and CG (*P*>0.05); however, HR and TT were greater for the NC than the CG condition. The HR and TT values significantly were lower for the CV compared to the NC and CG conditions (*P*<0.05). The repeated measures analyses of variance also revealed there is no significant difference in HR and TT between the CV and CG+CV (*P*>0.05).

### Continuous performance test 

**Figure 6:**
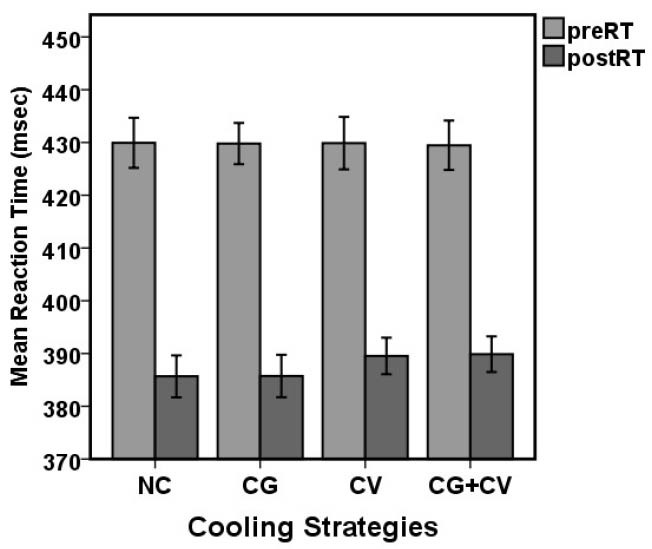
Comparison of the mean RT between the before (preRT) and after (postRT) activity in the 4 experimental conditions (*P*<0.05).
Abbreviations: NC, no cooling device; CG, cooling gel contain menthol; CV, cool vest; CG+CV, cooling gel and cool vest.

Figure 6 illustrates the effect of simulated firefighting tasks on RT. Before and after the 4 experimental conditions, mean RT scores were (429.93 ms [SD 8.59] vs. 385.67 ms [SD 7.19]), (429.80 ms [SD 7.06] vs. 385.73 ms [SD 7.25]), (429.87 ms [SD 8.98] vs. 389.53 ms [SD 6.24]), (429.47 ms [SD 8.45] vs. 389.87 ms [SD 6.12]), for NC, CG, CV and CG+CV, respectively. Firefighters showed faster RT relative to baseline throughout the exercise condition (*P*<0.05).

**Figure 7:**
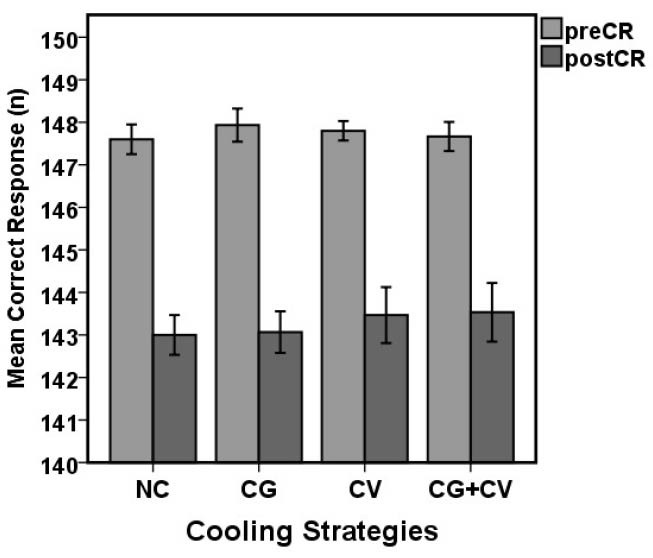
Comparison of the mean CR between the before (preCR) and after (postCR) activity in the 4 experimental conditions (*P*<0.05).
Abbreviations: NC, no cooling device; CG, cooling gel contain menthol; CV, cool vest; CG+CV, cooling gel and cool vest.

Figure 7 demonstrates the effect of simulated firefighting tasks on CR. Before and after the 4 experimental conditions, mean CR scores were (147.60 [SD 0.63] vs. 143.00 [SD 0.84] ), (147.93 [SD 0.70] vs. 143.07 [SD 0.88]), (147.80 [SD 0.41] vs. 143.47 [SD 1.18] ), (147.67 [SD 0.84] vs. 143.53 [SD 1.24) ], for NC, CG, CV and CG+CV, respectively. CR decreased significantly relative to baseline during simulated firefighting activities (*P*<0.05).

Table 2 shows the pairwise comparisons of cooling impacts on cognitive function responses. A repeated measures analyses of variance was used for pairwise comparisons to examine the effect of cooling strategies on the cognitive function. As Table 2 indicates, there is no significant difference in RT and CR between NC and CG (*P*>0.05). The values for RT and CR were higher for the CV compared to the NC and CG condition (*P*<0.05). The repeated measures analyses of variance also showed no significant difference in RT and CR between the CV and CG+CV conditions (*P*>0.05).

## Discussion

The aim of the present study was to investigate the effectiveness of cooling strategies on cognitive function and physiological strain during firefighting activities while wearing protective clothing in the smoke-diving room. It was hypothesized that the application of a cooling strategy would ameliorate cognitive function and reduces physiological strain following moderate-intensity activities in the smoke-diving room.

The results of the current study are in agreement with previous studies, which have shown that the combined effects of strenuous activities during firefighting in hot environments while wearing FPC induce notable physiological strain. According to Faff and Tutak,^35^ HR and rectal temperature values increased significantly throughout the exercise. In the present study, physiological responses especially HR responses are similar to those reported during simulated firefighting.^20,36^

Like former research, the practical cooling strategies employed in the present study were useful in attenuating physiological responses during simulated firefighting in the smoke-diving room.^11,20,21,26^Chou et al,^26^ reported that using phase change material cooling devices is more effective than using ice-pack cooling devices in reducing mean skin and rectal temperature during exercise while wearing FPC. According to Barr et al,^20^ core temperature, HR, and mean skin temperature were notably lower following the recovery time in the cooling condition compared with the control one.

In the current study, at the end of the firefighting activity, HR and TT increased in the NC, CG, CV and CG+CV conditions. The notably increased HR and TT responses that were evident in the first (NC) condition during the firefighting task’s period were not observed during the other experimental condition. HR is a physiological response closely associated with physical activity and heat stress. It is considered a rapid physiological response to physical activity and heat.^13,37^The findings of this study showed that there are differences in attenuating physiological load among cooling strategies; the CV is much easier to use in real status of firefighting work. In addition, CV does not require refrigeration. These results demonstrate that cooling vest is more effective than CG in attenuating the physiological load during firefighting activity while wearing FPC. In the current study, the application of CV in combination with the CG was not effective in attenuating HR and TT compared to the application of cooling vest method alone during the firefighting activity. Therefore, combining cooling vest with CG application provides no additional benefit.

While extinguishing fire, firefighters need to work in hot, smoky environments for a long time with few or no breaking times. Prior research has indicated that insularity, heat, smoke, and sleep deprivation can have detrimental effect on cognitive function and physical capacities.^38^ Research indicates that simulated firefighting would have a considerable effect on cognitive function.^2,24,39^ Previous study investigated stress reactivity and cognitive function in a simulated firefighting task and reported cognitive function impairments following a simulated firefighting.^9^ Kivimäki and Lusa,^40^ investigated the relationship between stress reaction and cognitive function during simulated firefighting, on the one hand, and an unknown rescue task in the dark labyrinthine, on the other hand. The results showed that as the stress reaction during simulated smoke-diving task increased, the controlled task-focused thinking declined. They assessed firefighters’ stress reactions in the light of alterations in their rest and maximal HR during the simulated exercise.

Based on this research, it is supposed that body cooling strategies would modulate decrements in cognitive function during firefighting activities while wearing FPC. The ability to keep sustained attention and rapidly detect threats is significant in a hostile environment; that is, firefighters need to react instantly to intervene and take suitable actions and remember main details such as critical geographical positions to conduct search and rescue. Previous studies have employed CPT test for the assessment of cognitive functions, including sustained attention and information processing during simulated firefighting activities.^2,39,41^

McMorris et al,^42^ discovered that when plasma norepinephrine concentrations go up, number of errors committed on a flanker task during heavy exercise increases too; however, the greater the increases in epinephrine and adrenocorticotropic hormone (ACTH) concentrations, the better the response time. Since norepinephrine, epinephrine, and ACTH are indicative of increased arousal, it may be hypothesized that the speed and accuracy components of the cognitive function task were influenced in different ways. The increase of neurotransmitters may result in higher speed of processing; nevertheless, neural noise could have negative influence on the accuracy of completing cognitive function task.^42^ In the current study, firefighters’ cognitive function impairments were significantly greater after simulated firefighting activities compared to those prior to the exercise. RT was faster and CR was lower at the end of firefighting activities for all trials. As mentioned above, the CPT is exploited to measure cognitive function. It is thus argued that decreases in CPT scores after being involved in firefighting activity indicate impairment in cognitive function. Comparison of mean changes in RT and correct answer showed differences between the 4 experiments. According to the results, cognitive function impairment was higher for NC experiment at the end of firefighting activities. CG containing menthol treatments did not affect the cognitive function compared to the NC condition. But the CV caused decreases in errors in the CPT test. These results demonstrate that cooling vest is more effective than CG containing menthol in reducing the cognitive function impairments during firefighting activity. It is worth mentioning that combining cooling vest with CG containing menthol was not effective in attenuating cognitive function impairment compared to the application of cooling vest alone, during the firefighting activity period. This is in agreement with the findings involving a group of professional firefighters in which change in RT and choice accuracy were detected following a firefighting simulation.^12,24,39^ In addition, the results of the present study are backed by the findings of a previous study, which reported that menthol treatment did not significantly affect the mood, RT, and response accuracy compared to the placebo (*P*>0.05).^28^ Furthermore, Lee et al,^24^ studied neck cooling and cognitive function following exercise on a treadmill at 70% VO2 peak under warm and humid conditions (dry bulb temperature: 30.2 ± 0.3^°^C, RH: 71 ± 2%) for 75 minutes or until the subjects experienced volitional fatigue. They reported that neck cooling may enhance cognitive function following exercise.

### Advantages and limitations of the present study

In the current study, professional and experienced firefighters were recruited. They conducted simulated firefighting activities in an advanced and well-equipped smoke-diving room (e.g. nesting and dark rooms, heating and cooling systems, specialized UV cameras, and artificial smoke and steam generators) that resembles real firefighting scenarios. Furthermore, practical cooling procedures (i.e. CG and CV), which do not need any cooling, freezing, and preparing process, were exploited to reduce individuals’ physiological strain and cognitive dysfunctions during the firefighting activities. Nevertheless, we could not simulate the radiant heat of direct fire exposure in our smoke-diving room since it has insignificant effect on typical firefighting and rescue operations far from the fire scene.^11,43,44^ Instead, the defined activities in the simulated activity are the most typical and most challenging firefighting tasks.^44,45^ Moreover, it should be noted that many firefighting tasks do not involve direct exposure to a fire but still require wearing FPC and SCBA.^11^ Nevertheless, further research will be needed to compare the effect of real fire suppression with typical firefighting tasks on firefighters’ physiological response and cognitive function. Furthermore, future prospective studies are needed to determine whether the practical cooling tactics can modulate decrements in physiological response and cognitive function during the real fire extinguishing.

## Conclusion

It is concluded that strenuous and multiple duties during fire and rescue operations have a detrimental effect on firefighters’ physiological response and cognitive function. The findings also revealed that CV coated with phase change material was more effective than the CG containing menthol in attenuating physiological responses and cognitive function during firefighting and life-saving operations. Moreover, combining cooling vest with CG application provides no additional benefit. It can be concluded that, cooling the body using CV offered physiological and cognitive functions benefits for firefighters during simulated firefighting activities.

## Ethical approval

The study was approved by Vice-chancellor for Research and Technology, Hamadan University of Medical Sciences (No. 9504222161). All participants signed the consent form and confidentiality of data collection was ensured to all of them.

## Competing interests

None of the authors has any conflict of interest.

## Authors’ contributions

MM and MA conceptualized the study, designed data collection methodology, and led manuscript development. RH collected all the data and drafted the first draft of the manuscript. OK contributed to the writing of the manuscript. MF conducted data analyses.

## Acknowledgments

The authors would like to appreciate the financial backing provided by Hamedan University of Medical Sciences and would like to thank the head of Health, Safety and Environment (HSE) department of Iran’s National Petrochemical Company (NPC) who helped us to perform the study. This study has been adapted from a Ph.D. thesis at Hamadan University of Medical Sciences.

**Table 1:** Pairwise comparison of the effectiveness of cooling strategies on the physiological responses

**Cooling strategies**	**HR (bpm)**				**TT (** ^○^ **C)**			
	**MD**	**95% CI**		***P*** ** value**	**MD**	**95% CI**		***P*** ** value**
		**Lower**	**Upper**			**Lower**	**Upper**	
NC vs. CG	0.73	-0.190	1.657	0.111	0.01	-0.018	0.038	0.458
NC vs. CV	6.86	4.950	8.783	<0.001	0.20	0.147	0.260	<0.001
NC vs. CG+CV	6.93	5.491	8.375	<0.001	0.23	0.134	0.326	<0.001
CG vs. CV	6.13	3.920	8.347	<0.001	0.19	0.153	0.234	<0.001
CG vs. CG+CV	6.20	4.673	7.727	<0.001	0.22	0.138	0.302	<0.001
CV vs. CG+CV	0.06	-1.030	1.164	0.898	0.02	-0.031	0.084	0.334

Abbreviations: HR, heart rate; TT, temporal temperature; MD, mean difference; CI, confidence interval for difference; NC, no cooling device; CG, cooling gel contain menthol; CV, cool vest; CG+CV, cooling gel and cool vest.

**Table 2:** Pairwise comparison of the effectiveness of cooling strategies on the cognitive function responses

**Cooling strategies**	**RT (ms)**				**CR (n)**			
	**MD**	**95% CI**		***P*** ** value**	**MD**	**95% CI**		***P*** ** value**
		**Lower**	**Upper**			**Lower**	**Upper**	
NC vs. CG	0.06	-0.395	0.262	0.670	0.06	-0.210	0.076	0.334
NC vs. CV	3.86	0.893	6.841	0.014	0.46	0.112	0.821	0.014
NC vs. CG+CV	4.20	1.187	7.213	0.010	0.53	0.122	0.945	0.015
CG vs. CV	3.80	0.729	6.871	0.019	0.40	0.050	0.750	0.028
CG vs. CG+CV	4.13	1.023	7.244	0.013	0.46	0.112	0.821	0.014
CV vs. CG+CV	0.33	-0.663	1.330	0.485	0.06	-0.076	0.210	0.334

Abbreviations: RT, reaction time; CR, correct response; MD, mean difference; CI, confidence interval for difference; NC, no cooling device; CG, cooling gel contain menthol; CV, cool vest; CG+CV, cooling gel and cool vest.
